# Catalytic green synthesis of Tin(IV) oxide nanoparticles for phenolic compounds removal and molecular docking with EGFR tyrosine kinase

**DOI:** 10.1038/s41598-024-55460-4

**Published:** 2024-03-19

**Authors:** S. F. Alshahateet, R. M. Altarawneh, W. M. Al-Tawarh, S. A. Al-Trawneh, S. Al-Taweel, K. Azzaoui, M. Merzouki, R. Sabbahi, B. Hammouti, G. Hanbali, S. Jodeh

**Affiliations:** 1 Department of Chemistry, Faculty of Science, Mutah University, Al-Karak, Jordan; 2 Engineering Laboratory of Organometallic, Molecular Materials and Environment, Faculty of Sciences, Sidi Mohamed Ben Abdellah University, 30000 Fez, Morocco; 3 Euro-Mediterranean University of Fes, BP 15, 30070 Fez, Morocco; 4 Morocco Laboratory of Applied Chemistry and Environment (LCAE) Team (ECOMP), Mohamed 1er University, Oujda, Morocco; 5 Higher School of Technology, Ibn Zohr University, P.O. Box 3007, Laayoune, Morocco; 6 Department of Chemistry, An-Najah National University, Nablus, Palestine

**Keywords:** Green synthesis, SnO_2_ NPs, *Ocimum basilicum*, Adsorption, Phenol, *p*-Nitrophenol, *p*-Methoxyphenol, Nanoparticles, Kinetic, Isotherm, Thermodynamic, Molecular docking, Environmental chemistry, Environmental impact, Environmental sciences, Chemistry

## Abstract

In this study, tin dioxide nanoparticles (SnO_2_ NPs) were successfully synthesized through an eco-friendly method using basil leaves extract. The fabricated SnO_2_ NPs demonstrated significant adsorption capabilities for phenol (PHE), *p*-nitrophenol (P-NP), and *p*-methoxyphenol (P-MP) from water matrices. Optimal conditions for maximum removal efficiency was determined for each phenolic compound, with PHE showing a remarkable 95% removal at a 3 ppm, 0.20 g of SnO_2_ NPs, pH 8, and 30 min of agitation at 35 °C. Molecular docking studies unveiled a potential anticancer mechanism, indicating the ability of SnO_2_ NPs to interact with the epidermal growth factor receptor tyrosine kinase domain and inhibit its activity. The adsorption processes followed pseudo-second order kinetics and Temkin isotherm model, revealing spontaneous, exothermic, and chemisorption-controlled mechanisms. This eco-friendly approach utilizing plant extracts was considered as a valuable tool for nano-sorbent production. The SnO_2_ NPs not only exhibit promise in water treatment and also demonstrate potential applications in cancer therapy. Characterization techniques including scanning electron microscopy, UV–visible spectroscopy, Fourier transform infrared spectroscopy, X-ray diffraction spectroscopy (XRD), and energy-dispersive X-ray spectroscopy (EDAX) provided comprehensive insights into the results.

## Introduction

Advancements in human, industrial, and agricultural pursuits bring numerous benefits, yet concurrently contribute to heightened pollution, particularly in aquatic ecosystems. These contaminants cause damage to the health of organisms and the environmental level in general. In this regard, many countries limited themselves to determining the lowest concentration of these pollutant with legal obligations. However, protecting water resources and improving new technologies for water treatment have become critical environmental concerns and severe challenges of the twenty-first century^1^.

Chemical pollutants are typically of organic origin^2^. Among them, phenolic compounds are a class of the most common pollutants in wastewater. In general, these compounds are a common class of aromatic hydrocarbons. They mainly comprise a benzene ring bonded with the hydroxyl group (OH). Such compounds are a priority, very soluble in water, less volatile, and low bio-degradability. They can be found in the environment as raw materials, intermediate compounds, or final products^3^.

Moreover, phenolic compounds are also toxic to humans, plants, animals, and aquatic life^4^. Unfortunately, these compounds are abundantly found in high concentrations in different water systems, such as pesticides, plastics, textiles, pigments, and chemical residues^4,5^. The concentration of phenolic compounds in aqueous wastes can reach hundreds of milligrams per liter^6^. Therefore, phenolic compounds are among the most prominent organic pollutants.

Removal of phenolic hydrocarbons from water is a considerably tricky process. So, many treatment techniques were performed for water purification^7^. Among these techniques, adsorption technology is widely used to remove several organic pollutants from water due to its exceptional features such as simplicity, economics, flexibility, and effectiveness. For this purpose, various adsorbents were used to isolate phenol and substituted phenolic pollutants, such as activated carbon, supermolecular compounds, biological waste, metal oxides, and nanoparticles^8–11^.

Nanotechnology is making a great influence on several novel applications^12,13^. Nanoparticles are one of the most significant examples of nanotechnology applications. That is attributed to their unique characteristics, such as extremely small size and large surface area^14,15^. Some common types of nanoparticles include metal oxide nanoparticles, quantum dots, carbon nanotubes, and liposomes. Nanoparticles typically show unique chemical, physical, and biological properties that differ from their bulk (normal-scaled) counterparts^16^. However, it is important to observe that while several nanoparticles offer many promising benefits, their effect on living organisms health and the environment is also a topic that needs advanced research, so regulation and risk evaluation are essential to ensure their safe use. Herein, Tin(IV) Oxide nanoparticles (SnO_2_ NPs) are among the first inorganic (especially metal oxide) nanoparticles to be examined as a potential adsorbent surface.

The basil, *Ocimum basilicum*, a common herb of the Lamiaceae family known for its ornamental and therapeutic importance, was used in this work for the eco-friendly synthesis of SnO_2_ NPs. *O. basilicum* leaves have strong antioxidants and antimicrobial activity, in addition to therapeutic importance for respiratory and digestive issues^17–19^. By using this herb as a natural raw material in eco-friendly synthesis processes, fewer chemical substances can be employed that are harmful to the human. Meanwhile, these nanoparticals contribute significant improvements to global environmental sustainability through the use of renewable resources and the reduction of waste generation^5,6^. Nanoparticles fabricated from *O. basilicum* exhibit the potential to enhance the quality of water through the removal of heavy metals and organic contaminants^15,20,21^.

This study aims to synthesize the SnO_2_ NPs using *O. basilicum* leaves extract. The SnO_2_ NPs were also screened for the removal of phenol, *p*-nitrophenol, and *p*-methoxyphenol molecules from water systems in various experimental parameters. Furthermore, the study presents a detailed examination of the adsorption capacities, equilibrium, kinetics, isotherm, and thermodynamics interpretation. Moreover, molecular docking studies have also been carried out to reveal the potential of SnO_2_ NPs to interact with the epidermal growth factor receptor (EGFR) tyrosine kinase domain and inhibit its activity, suggesting a novel anticancer mechanism of action. The eco-friendly approach applied here will serve as a valuable tool for the production of nano-sorbents based on plant extracts, and SnO_2_ NPs have promising applications in water treatment and anticancer therapy.

## Methodology

### Materials

All chemicals were used as-received, and deionized water (D.H_2_O, 18.2 µΩ cm^−1^) was used to prepare all solutions / suspension. All phenol (99.0%), *p*-Nitrophenol (99%), and *p*-Methoxyphenol (99.0%), were purchased from Sigma Aldrich. Tin(IV) Chloride (SnCl_4_.5H_2_O, 99%) was purchased from Gainland Chemical Company. Ethanol (C_2_H_6_O, 99.5%) was obtained from Merck. The fresh leaves of *O. basilicum* were collected from the Al-Karak area, Jordan, and taxonomically identified by the Department of Biological Sciences, Faculty of Science, Mu'tah University, Al-Karak, Jordan.

### Instrumentation and characterization*.*

A pH meter (HANNA instruments, HI5521-02, UK) was employed to estimate the pH solutions. The orbital shaker (LAUDA, Germany) was used to shake solution contents. A hot plate (Bibby Scientific HB502, UK) was utilized to raise the temperature of the adsorption system. A UV–vis spectrophotometer (Perkin-Elmer Model lambda 25, USA) was utilized to analyze the residual phenolic compound content employing a 1 cm path length quartz cuvette (Hellma, Germany). The tin oxide (SnO_2_ NPs) was monitored by UV–visible spectroscopy (Schimadzu UV–visible spectrophotometer, model UV-1800). More characterization was done using powder XRD-analysis (Advance Powder X-ray diffractometer, Bruker, Germany, model D8), The characterization of peaks for the Fourier transform-infrared spectroscopy (FT-IR) was done using (Alpha TBruker), for the morphological studies for the surface was done by SEM (Hitachi H-7100 using an accelerating voltage of 120 kV). The elemental analysis was studied using EDAX (Bruker, Germany).

### Formation of O. Basilicum Leaves Extract

The *O. basilicum* leaves were washed with tap water to remove any dust particles, then they were rinsed with D.H_2_O and were thoroughly dried in the shade at ambient temperature. To prepare the extract, 20 g of the clean and dry leaves were added into an air-tight container with 200 ml of boiled D.H_2_O for 90 min. After letting the mixture come to ambient temperature and filtering it, centrifugation was performed for 20 min at 4000 rpm. The final greenish-yellow extract was kept at 4 °C for further experiment.

### Fabrication of SnO_2_ NPs

**Figure 1:**
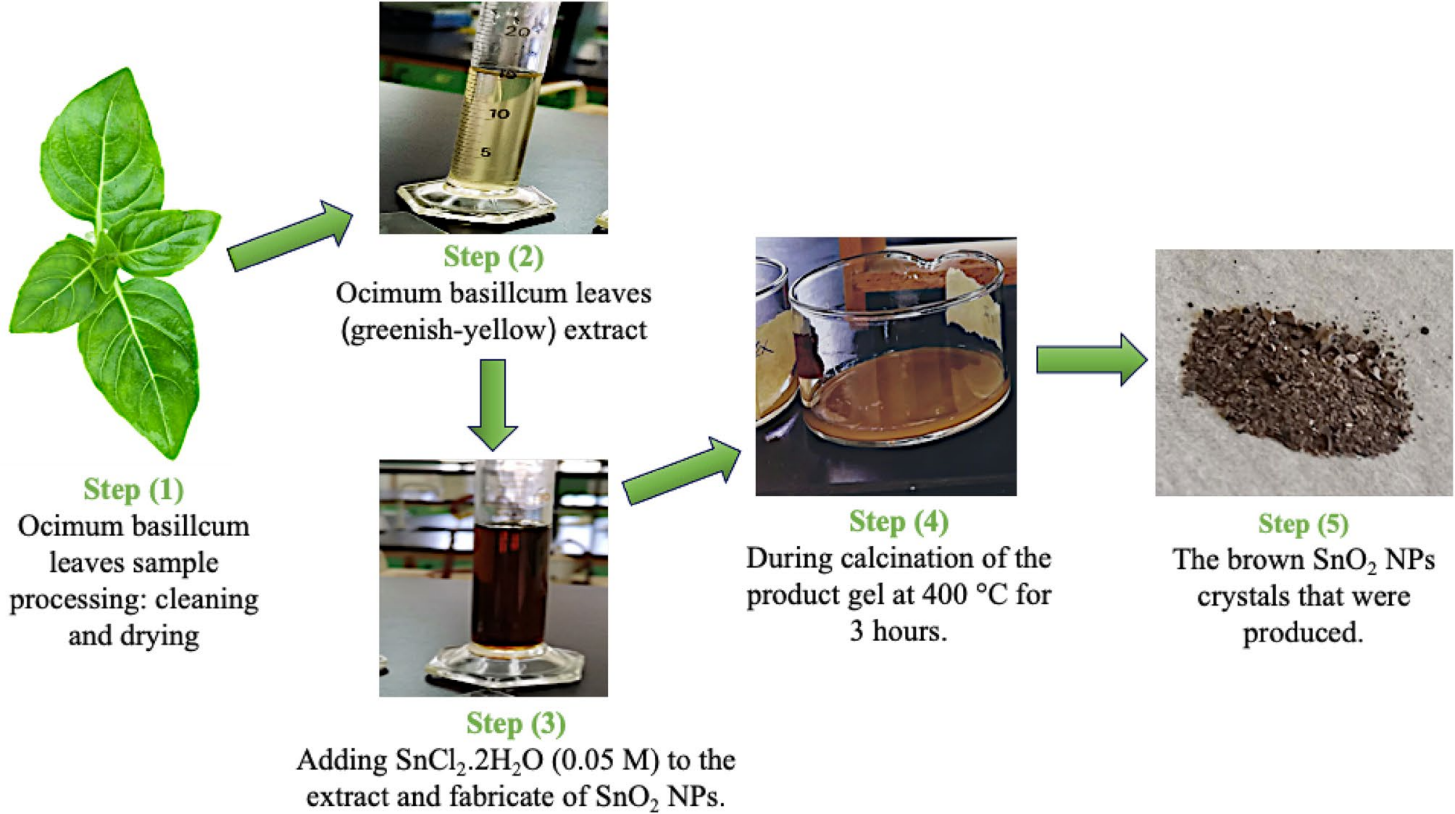
Schematic representation of the fabrication of SnO_2_ NPs using the *Ocimum basilicum* extract.

The target SnO_2_ NPs were prepared by mixing 6.25 ml of SnCl_4_.5H_2_O (0.05 M) with 6.25 ml of *O. basilicum* leaves extract while heating (80 °C) and stirring (250 rpm) continuously for 20 min. The aged gel was thoroughly washed three times using hot D.H_2_O, stirring for 2 min, and letting it settle for sufficient time. The product was centrifuged (20 min at 3000 rpm) and washed again with ethanol. After that, it was calcined at 400 °C for 3 h. The resulting brown crystals were finely crushed into a powder and stored in a polyethylene bottle for subsequent use in batch adsorption experiments. Figure 1 depicts a schematic illustration of the green synthesis route for producing SnO_2_ NPs using *O. basilicum* leaves extract.

### Batch adsorption experiments

The adsorption experiments of phenolic compounds on fabricated SnO_2_ NPs were carried out using batch adsorption techniques. All experiments used Aluminum foil to wrap 150 mL Erlenmeyer flasks to prevent light effects on phenolic solutions. Several operational parameters; adsorbent dosage (0.10, 0.15, 0.20 and 0.30 g), agitation time (5, 15, 30, and 60 min), starting adsorbate concentration (1, 3, 7 and 10 ppm), pH (4, 6, 8 and 10) and temperature (25, 35, 45 and 55 °C) were all examined and adjusted as factors affecting by the PHE, P-NP, and P-MP adsorption process.

The batch experiments were performed by mixing fabricated SnO_2_ NPs powder and phenolic solutions in different ratios (% w/w) in a 50 mL aqueous media and equilibrated at an orbital shaker for different agitation times at constant shaking speed (240 rpm). After that, all samples were centrifuged, and the absorbent went through a 0.22 μm filter membrane. The phenolic content residue in supernatants was analyzed using a UV–Vis spectrophotometer before and after adsorption. To understand the impact of adsorbent and adsorbate dosage, the adsorbent and phenolic solutions were mixed in different ratios by varying the formed SnO_2_ NPs dosage from 0.10 to 0.30 g and phenolic solutions starting concentration from 1 to 10 ppm under optimal conditions. The initial pH of each phenolic solution was rigorously adjusted from 4 to 10 using hydrochloric acid (HCl, 1 M) and sodium Hydroxide (NaOH, 1 M) solutions. Different agitation times from 5 to 60 min were prepared to determine the optimum agitation time between SnO_2_ NPs and phenolic solutions. The removal efficiency and adsorption capacity are calculated from Eqs. (1) and (2), respectively. Where the starting (C_i_) and final (C_t_) amounts of phenolic compounds as well as the mass of SnO_2_ NPs (*M*), and the volume of the aqueous media used (*V*)^22^. All batch adsorption experiments were conducted in triplicate (n = 3), and the average results were provided.

1 $${\text{Removal}}\,{\text{efficiency}}\,(\% ) = \frac{{{\text{C}}_{{\text{i}}} - {\text{C}}_{{\text{f}}} }}{{{\text{C}}_{{\text{i}}} }} \times 100$$

2 $${\text{Adsorption}}\,{\text{capacity}}\,({\text{mg}}/{\text{g}}) = \frac{{\left( { {\text{C}}_{{\text{i}}} - {\text{C}}_{{\text{f}}} } \right) }}{{\text{M}}} \times {\text{V}}$$

### Kinetics, isotherm, thermodynamic analysis

To further understand the mechanism of PHE, P-NP, and P-MP adsorption process progressed, several adsorptive characteristics of fabricated SnO_2_ NPs were diagnosed in this study. The kinetic was studied by estimating the adsorption rate upon arrival for equilibrium. The adsorption isotherm mechanism was analyzed using the Langmuir, Freundlich, and Temkin isotherm models to explore the relationship between the SnO_2_ NPs capacity and the phenolic concentration in the surrounding solution upon equilibrium. In the thermodynamic context, the significant parameter of the Gibbs free energy changes (ΔG^0^), the standard enthalpy changes (ΔH^0^), the standard entropy changes (ΔS^0^), and the distribution coefficient (*K*_*d*_) were calculated and interpreted.

### Molecular docking and protein preparation

The Molecular Operating Environment (MOE) software was employed for the assessment of the binding capabilities of specific SnO_2_ NPs obtained from the PubChem database (http://pubchem.ncbi.nlm.nih.gov). The 3D structure of these NPs was prepared in an SDF (structure-data file) format and selected for subsequent molecular docking studies. Two distinct methods were utilized for the screening of compounds: (a) pharmacophore-based screening and (b) molecular docking. In the pharmacophore-based virtual screening, the Compute option within MOE was used to identify the pharmacophoric features of the co-crystalized ligand. Following the selection of these ligand features, the software was executed to filter compounds based on the identified features within the co-crystal ligand. The resulting output file containing the selected compounds was then employed for subsequent molecular docking studies^19,23^.

In employing a molecular docking strategy, the ligands, pre-screened based on pharmacophore considerations, were subjected to docking with the 3D structure of the target protein. The investigation focused on determining the most favorable outcome in terms of biological activity, utilizing the crystal structure of the inactive EGFR tyrosine kinase domain in complex with erlotinib (PDB: 4HJO), with a resolution of 2.75 Å^24^. This crystal structure was retrieved from the Protein Data Bank (PDB) and meticulously prepared in the MOE for subsequent docking analyses. To anticipate the active site residues within the binding pocket, several steps were taken, including 3D protonation, energy minimization of the protein, and utilization of a site finder. The preparation of the protein structure involved tasks such as adding missing hydrogen atoms, correcting bond order assignments, adjusting charge states and orientations of various groups, and performing restrained minimizations that allowed hydrogen atoms to be freely optimized^25,26^.

### Resources

The study was in accordance with relevant institutional, national, and international guidelines and legislation.

## Results and discussion

### X-ray diffraction (XRD) analysis of SnO_2_ NP

To investigate the phase formation and purity of the sample, Xray diffraction technique is used. The X-ray diffraction pattern of synthesized SnO2 nanoparticles was recorded at a scanning rate of 1^0^/min and within scanning angle range of 20–70.

**Figure 2:**
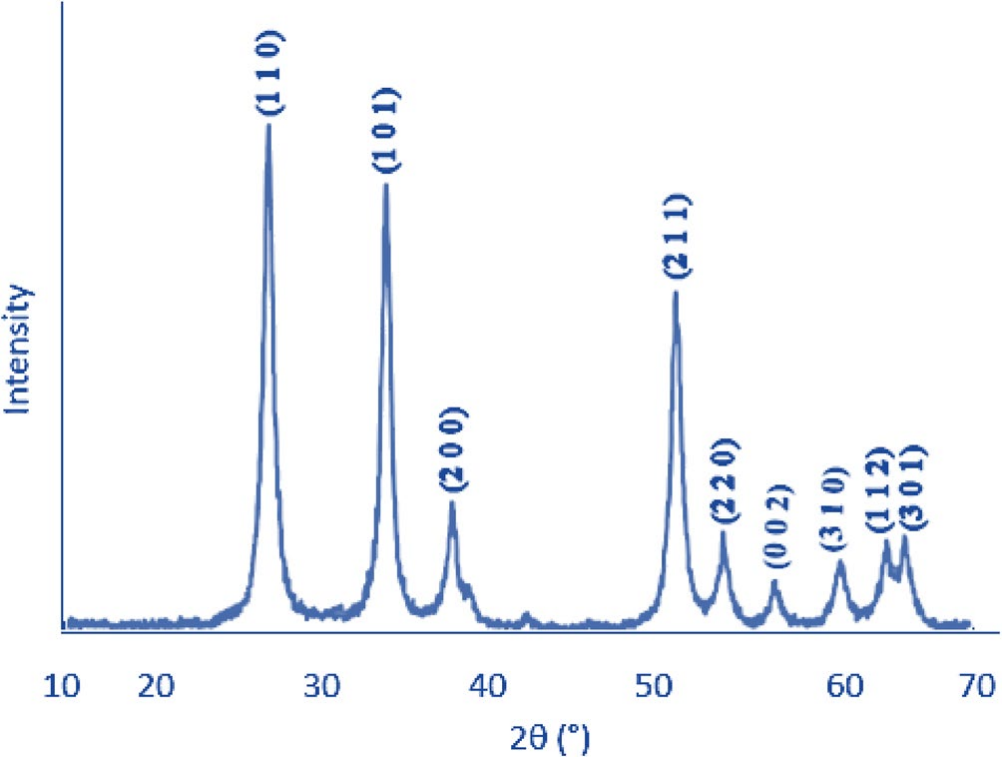
XRD spectrum of SnO2 nanoparticles.

XRD analysis for the prepared SnO_2_ NPs is shown in Fig. 2. The X-ray diffraction peaks of synthesized SnO_2_ NPs was made and plotted at a scanning rate of 2^0^/min with scanning angle range of 20^0^–80^0^. The diffraction peaks are observed at 2θ = 26.7°, 34.2°, 38.0°, 52.0°, 54.9°, 57.8°, 61.1°, 65.2° and 66.1° which they belong to SnO_2_ NPs.

The observation of intense peaks represents the high crystalline nature of the prepared sample with very high purity. Those peaks of the XRD analysis confirmed the type of crystalline SnO_2_ which was confirmed in the literature and by other researchers^27^. All the observed peaks are matched with standard JCPDS card No. 41-1445 having tetragonal unit cell.

### Fourier transform-infrared spectroscopy

**Figure 3:**
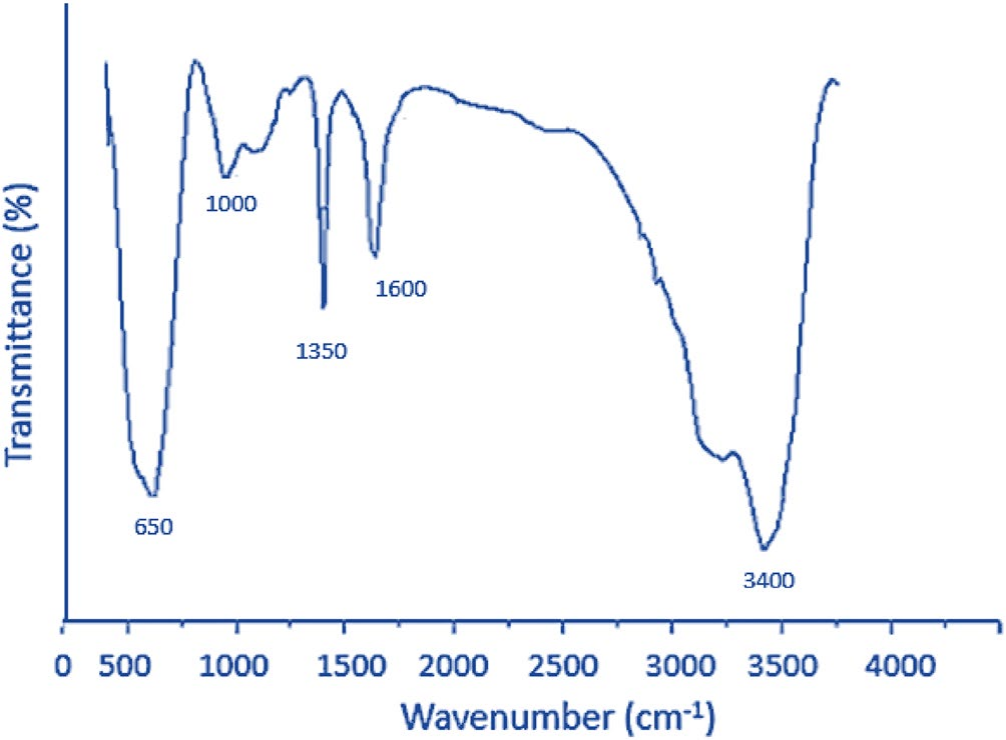
The Fourier Transform Infrared Spectroscopy (FT-IR) of the prepared SnO_2_ NPs.

The Fourier Transform Infrared Spectroscopy (FT-IR) of the prepared SnO_2_ is shown in Fig. 3. The FTIR shows stannous material with strong vibration in the range of 2600 to 3600 cm^−1^ that proves the existence of O–H bond resulting from adsorbed water and Sn–OH functional groups of SnO_2_ NPs^28,29^. The other peak at around 650 cm^−1^ is an indication of the anti-symmetric Sn–O–Sn stretching condensation of adjacent surface hydroxy groups.

### Scanning electron microscope and EDAX

**Figure 4:**
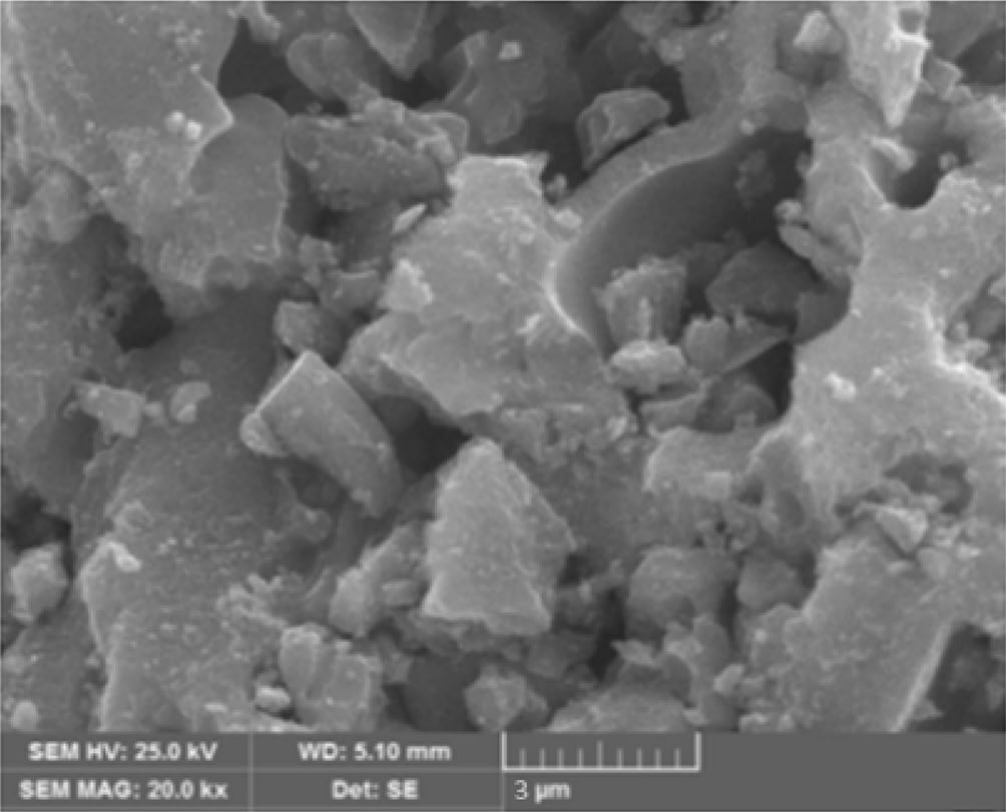
Scanning electron microscopy of SnO_2_ NPs.

**Figure 5:**
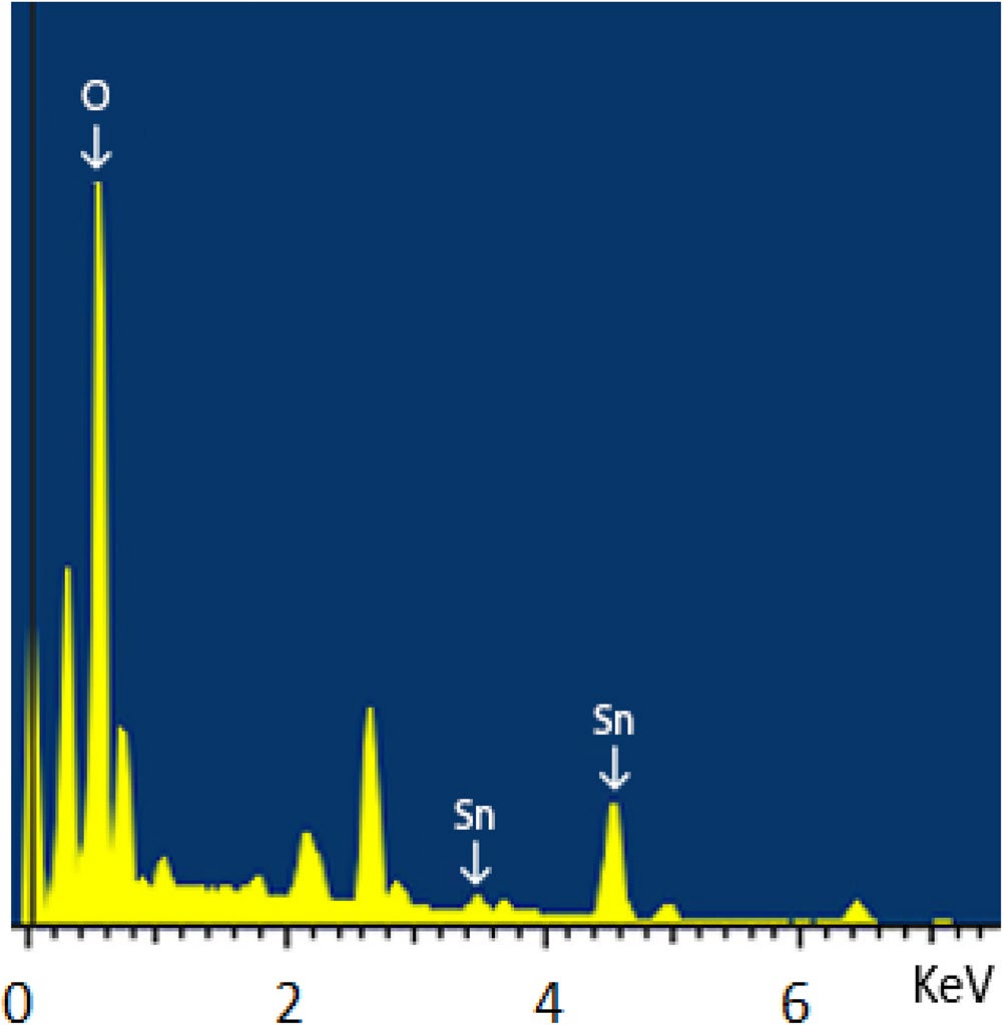
EDAX morphological analysis of SnO_2_ NPs.

The study of morphological characteristics of SnO_2_ NPs was studied using scanning electron microscopy (SEM) and the energy dispersive X ray analysis (EDAX). The SEM indicated the presence of very fine flakes that have very tiny agglomerates^19,27,29^ as shown in Fig. 4. The EDAX spectra showed that Sn and O are the most constituents of the SnO_2_ NPs as shown in Fig. 5.

### Optimization of operational parameters

#### Influence of nano-sorbent dosage

**Figure 6:**
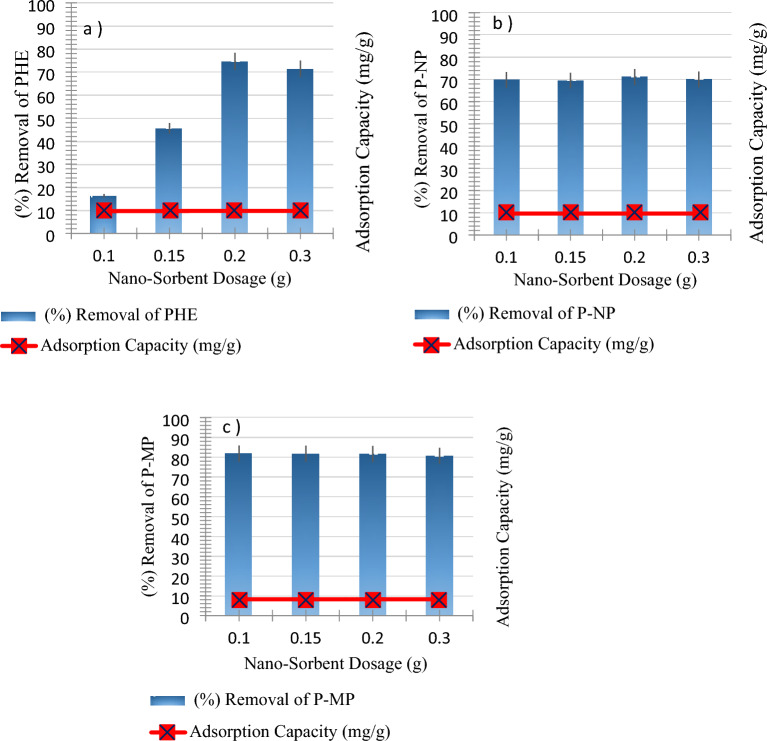
Impact of the nano-sorbent dosage on both adsorption capacity and removal efficiency for (**a**) PHE at pH = 8, (**b**) P-NP at pH = 8, and (**c**) P-MP at pH = 6 (Conditions: agitation time, 30 min; starting phenolic solution concentration, 10 ppm; at room temperature).

The impact of SnO_2_ NPs dosage on each PHE, P-NP, and P-MP adsorption onto SnO_2_ NPs surface was investigated with 50 mL of 10 ppm PHE, P-NP, and P-MP solutions for 30 min at 25 °C by using different nano-sorbent dosages of 0.10, 0.15, 0.20, and 0.30 g are shown in Fig. 6. Adsorption efficiency and capacity were calculated by using Eqs. (1) and (2). As depicted in Fig. 6a, the higher adsorption efficiency of PHE was 16, 46, 75, and 71% with 0.10, 0.15, 0.20, and 0.30 g of SnO_2_ NPs, respectively. This trend may be attributed to the active sites on the surface of phenol were initially unsaturated, but after reaching the best adsorption, the accumulation of adsorbents and competing substances causes adsorption impairment^30^. In contrast, with 0.10 g of SnO_2_ NPs, the higher adsorption efficiency of P-MP and P-MP were 82 and 71%, respectively. The adsorption efficiency almost remained constant as the nano-sorbent dosage increased, as shown in Fig. 6b, c. This may be due to the fact that the nano-sorbent has high surface areas relative to their very small size, this provides more space for adsorption even with the use of few dosages. However, increasing the nano-sorbent dosage may cause SnO_2_ NPs aggregation, thus impeding adsorption efficiency. In addition, it was observed that the adsorption capacity (amount) for PHE, P-NP, and P-MP were 9.72, 9.63, and 8.28 mg/g, respectively. The adsorption capacity remained constant as the nano-sorbent dosage decreased; this suggests a preference for using NPs as absorbent surfaces of target phenolic compounds^10^. Therefore, the nano-sorbent dosage was taken as 0.20 g of PHE, whereas 0.10 g of P-NP and P-MP in further experiments.

#### Influence of agitation time

**Figure 7:**
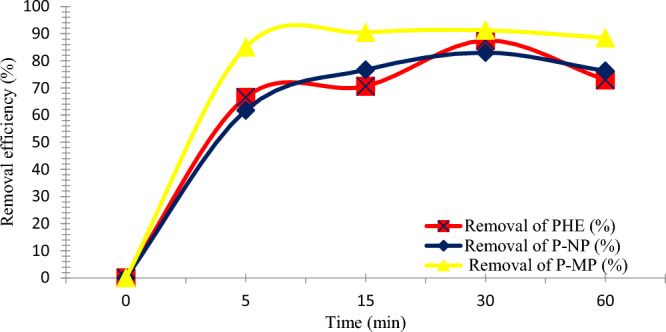
Impact of the agitation time on removal efficiency for PHE, P-NP, and P-MP (Conditions: nano-sorbent dosage, PHE (0.20 g), P-NP, and P-MP (0.10 g); starting phenolic solution concentration, 3 ppm; at 25 °C).

In order to evaluate the effect of agitation time on the adsorption uptake of PHE, P-NP, and P-MP molecules, experiments were carried out under agitation times from 0 to 60 min. Figure 7 illustrates the removal efficiency of PHE, P-NP, and P-MP onto SnO_2_ NPs as a function of agitation time. The removal process occurred in two stages: a rapid removal within the first 5 min and a relatively slow adsorption within 5–60 min. The PHE, P-NP, and P-MP adsorption rates reached equilibrium at 30 min with 88, 83, and 91% removal effectiveness, respectively. These results could be attributed to the presence high number of vacant active sites and the appropriate pore size of the nano-sobent surface, which can promote the internal mass transfer and adsorption process progress. However, the adsorption rate decreased progressively within 30–60 min due to accumulated adsorbate on the adsorbent surface and a rise of internal diffusion resistance^28,31^. The results indicate SnO_2_ NPs prefer to remove PHE, P-NP, and P-MP molecules.

#### Influence of starting phenolic solutions concentration

**Figure 8:**
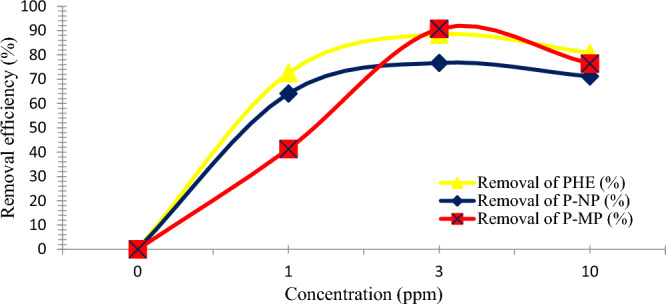
Impact of the starting phenolic solutions concentration on removal efficiency for PHE, P-NP, and P-MP (Conditions: nano-sorbent dosage, PHE (0.20 g), P-NP, and P-MP (0.10 g); agitation time, 30 min; at 25 °C).

The adsorption of PHE, P-NP, and P-MP onto the SnO_2_ NPs surface is highly dependent on the starting concentration of these phenolic solutions. This is related to the abundance of active sites on the nano-sorbent surface. Therefore, the impact of starting concentrations on adsorption quality was studied by varying PHE, P-NP, and P-MP concentrations from 1 to 10 ppm. The experiment was performed under the optimal parameters previously mentioned. The maximum removal efficiencies of each PHE, P-NP, and P-MP were obtained at 3 ppm of PHE, P-NP, and P-MP as a starting concentration, with 91, 88, and 77% removal, respectively. Afterward, adsorption efficiency decreased significantly as the starting concentration of the solution was increased, as depicted in Fig. 8. This phenomenon may attributed to the increased amount of PHE, P-NP, and P-MP ions with limited active sites on the SnO_2_ NPs surfaces, thus decreased the removal quality^32^. Moreover, this is a logical trend, as high concentrations of solutions are expected to be accompanied by increased agitation time required to achieve adsorption equilibrium. Our result agrees with previous literature^9,33^.

### Influence of the initial pH

**Figure 9:**
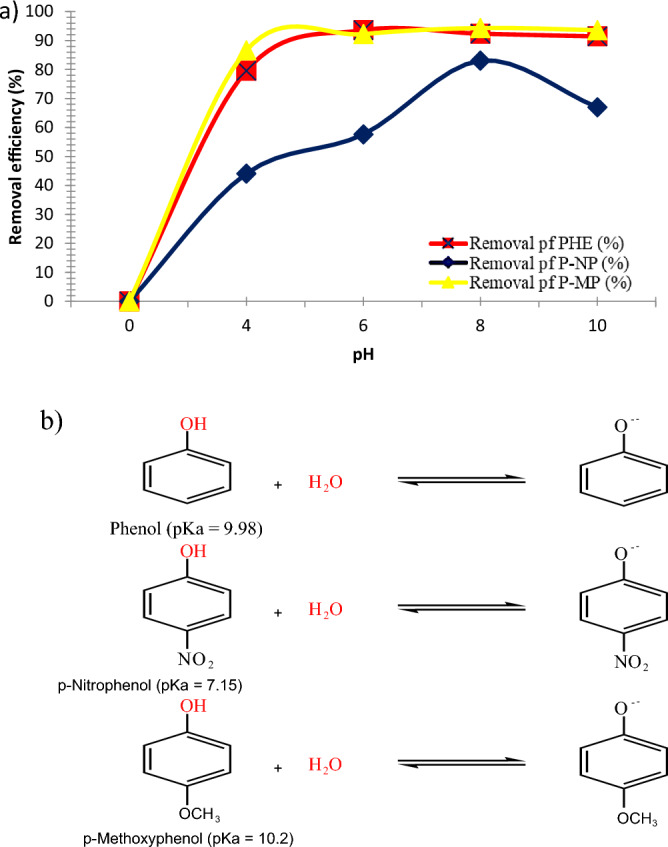
(**a**) Impact of the pH on removal efficiency for PHE, P-NP, and P-MP (Conditions: nano-sorbent dosage, PHE (0.20 g), P-NP, and P-MP (0.10 g), agitation time, 30 min; starting phenolic solutions concentration, 3 ppm; at 25 °C), and (**b**) Phenolate anions formation in aqueous media.

The pH of solutions constitutes a significant effect on the adsorption efficiency of phenols from aqueous systems as shown in Fig. 9. Particularly when the phenolic compounds may exist in various forms such as phenolic or phenolate relative to the pH of the solution. Therefore, the initial pH was studied to investigate its effect on the phenolic compounds adsorption at different initial pH values of 4, 6, 8, and 10, and starting phenolic solutions concentration of 3 ppm for 30 min at 25 °C. As illustrated in Fig. 9a, the maximum removal efficiency for PHE, P-NP, and P-MP were obtained at pH values of 8, 8, and 6 with 94, 94, and 83% removal, respectively. This phenomenon may be related to the pH_*ZPC*_ of SnO_2_ NPs, implying that the nano-sorbent surface has a zero charge at a specific pH value^8^. The adsorption efficiencies increased dramatically as the pH values increased. At a pH of lower than pHPzc, the nano-sorbent surface is positively charged. PHE, P-NP, and P-MP act as weak acids in the solutions, and all become phenolate anions, as depicted in Fig. 9b. Thus, the removal happens due to the dipole–dipole interaction at higher pH values. At much lower pH, additional positive protons compete for active binding sites on the nano-sorbent surface; this reduces adsorption efficiency. Conversely, at a pH of higher than pH_*ZPC*_, the nano-sorbent surface is negatively charged. Adsorption decreases slightly, and this may be due to the electrostatic repulsion interaction between the nano-sorbent and the adsorbate (negative charge holders). The achieved results agree with the previous literature's data^10^.

Furthermore, it can be seen that the effectiveness of the adsorption of both PHE and P-NP was greater than that of the adsorption of P-MP. This assessment is supported by the fact that the hydrogen binding capabilities of phenolic compounds are directly proportional to their acidities. The protons transfer to nano-sorbent surface is easier in the case of PHE and P-NP; this allows them to form H-bonds and achieve higher adsorption effectiveness. In the case of P-MP, weak H-bonding interactions are formed; this may be attributed to the steric effect of methoxy groups. Overall, a change in the solution pH predominantly impacts the strength of H-bonding, adsorbent-adsorbate interactions and adsorption efficiency^34^.

### Influence of temperature

**Figure 10:**
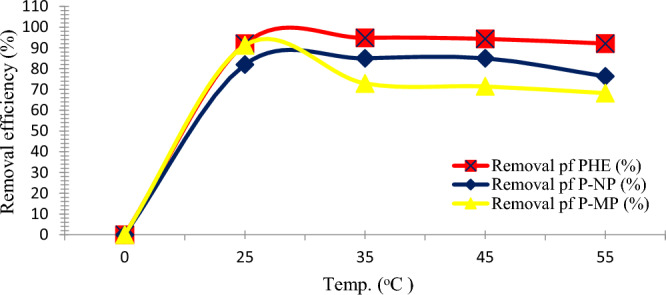
Impact of the temperature of adsorption system on removal efficiency for (**a**) PHE at pH = 8, (**b**) P-NP at pH = 8, and (**c**) P-MP at pH = 6; nano-sorbent dosage, PHE (0.20 g), P-NP, and P-MP (0.10 g), agitation time, 30 min; starting phenolic solution concentration, 3 ppm.

The impact of temperatures on each PHE, P-NP, and P-MP adsorption was assessed in this study. For this purpose, The adsorption studies were conducted at 25, 35, 45, and 55 °C, and optimized parameters were maintained during the experiment. The results demonstrated that the maximum adsorption effectiveness of P-MP was 94% at 25 °C, and then the adsorption significantly decreased as the temperatures rose to 68% at 55 °C, as shown in Fig. 10. This may refer to the destruction and damage of active binding sites on the SnO_2_ NPs surface due to high temperatures. It should be noted that the effectiveness of both PHE and P-NP removal increased significantly by increasing the temperature from 25 to 35 °C, then progressively decreasing as the temperature increased from 35 °C to 55 °C. Accordingly, it was determined that 35 °C is favorable for the PHE and P-NP adsorption onto SnO_2_ NPs with 95 and 85% removal, respectively. This result can be justified as it is with the increasing system temperature over 35 °C, the boundary's thickness will gradually drop. This can increase the tendency of PHE and P-NP to escape to the solution phase, decreasing adsorption^35^.

### Modelling of adsorption kinetics

**Figure 11:**
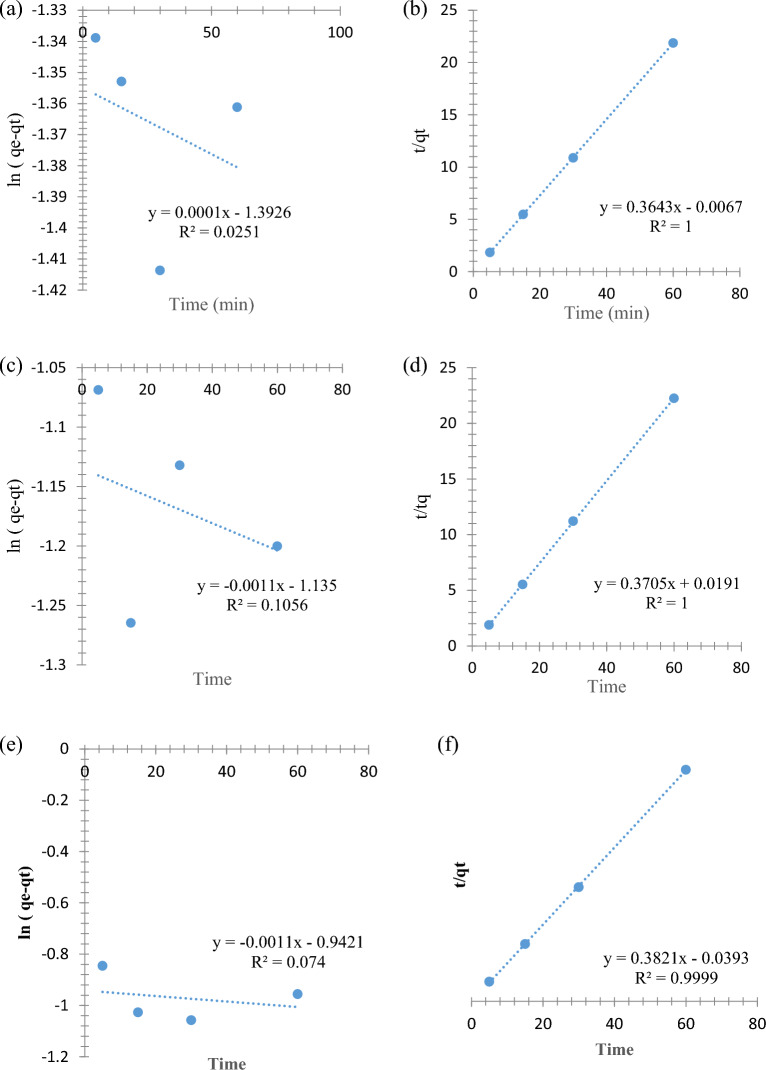
The kinetic plots of (**a**) pseudo-1st order for PHE removal, (**b**) pseudo-2nd order for PHE removal, (**c**) pseudo-1st order for P-NP removal, (**d**) pseudo-2nd order for P-NP removal, (**e**) pseudo-1st order for P-MP removal, and (**f**) pseudo-2nd order for P-MP removal.

The adsorption process of each PHE, P-NP, and P-MP was further characterized using the pseudo-1st order (Eq. 3) and pseudo-2nd order (Eq. 4) kinetic models and is shown in Fig. 11. The linearized forms of the pseudo-1st order (Fig. 11a,c, e) and pseudo-2nd order kinetic models are depicted in (Fig. 11b,d,f). The removal rate constants of the adsorption process were indicated by k_1_ and k_2_, respectively. Also, the adsorption quantity in mg of each PHE, P-NP, and P-MP per g of SnO_2_ NPs at both equilibrium (q_e_) and different agitation times (q_t_) was calculated from the adsorption experimental data.

3 $${\text{ln}}\left({q}_{e}-{q}_{t}\right)={\text{ln}}{q}_{e}-{k}_{1}t$$

4 $$\frac{{\text{t}}}{{{\text{q}}}_{{\text{t}}}}= \frac{1}{{{\text{k}}}_{2}{{\text{q}}}_{{\text{e}}}^{2}}+ \frac{{\text{t}}}{{{\text{q}}}_{{\text{e}}}}$$

The coefficient of determination (R^2^) value indicated that the adsorption of all PHE, P-NP, and P-MP more closely obeyed a pseudo-2nd order model. This model presumes the dominance of chemisorption over the adsorption mechanism of PHE, P-NP, and P-MP onto SnO_2_ NPs. This case involves exchanging or sharing electrons between the phenolates and SnO_2_ NPs^10,36^.

The experimental and calculated adsorption quantity (qe, mg/g) of PHE, P-NP, and P-MP at equilibrium and all kinetic parameters of pseudo-1st order and pseudo-2nd order models were calculated and presented in Table 1.

**Table 1:** Calculated kinetic parameters for PHE, P-NP, and P-MP adsorption.

Adsorbate	*q*_*e*, exp._ (mg/g)	Pseudo-1st order model			Pseudo-2nd order model		
		R^2^	K_1_ (min^−1^)	*q*_*e*, cal_ (mg/g)	R^2^	K_2_ (g min^−1^ mg^−1^)	*q*_*e*, cal._ (mg/g)
PHE	2.752	0.025	1.67 × 10^–6^	0.248	1	− 9.893	2.746
P-NP	2.718	0.106	− 1.83 × 10^–5^	0.321	1	7.187	2.699
P-MP	2.653	0.074	− 1.83 × 10^–5^	0.390	0.999	3.715	2.617

### Adsorption isotherms

**Figure 12:**
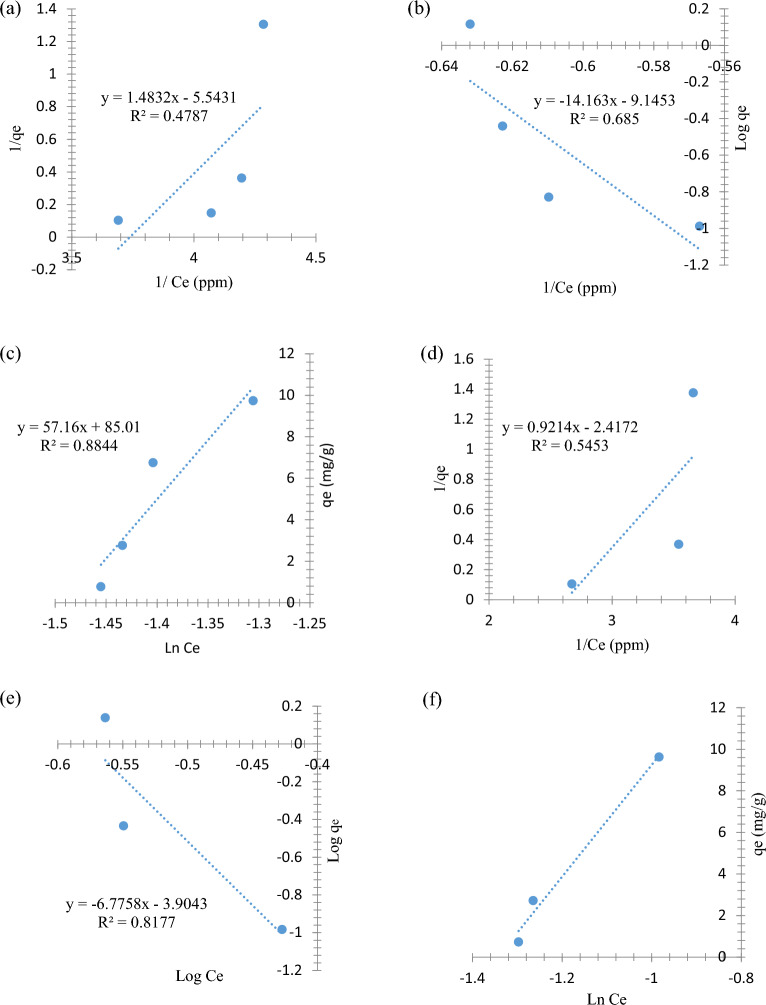

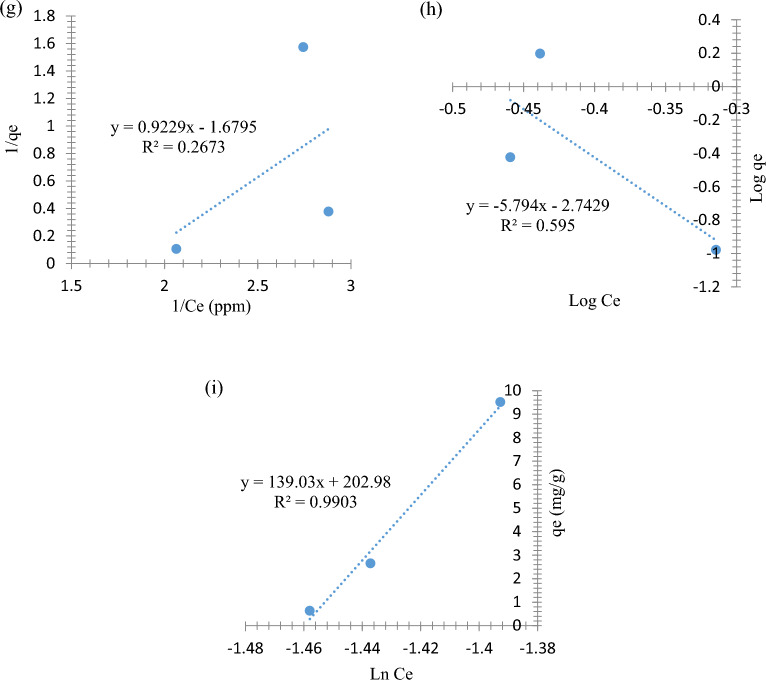
Isotherm plots of (**a**) Langmuir isotherm for PHE adsorption, (**b**) Freundlich isotherm for PHE adsorption, (**c**) Temkin isotherm for PHE adsorption, (**d**) Langmuir isotherm for P-NP adsorption, (**e**) Freundlich isotherm for P-NP adsorption, (**f**) Temkin isotherm for P-NP adsorption, (**g**) Langmuir isotherm for P-MP adsorption, (**h**) Freundlich isotherm for P-MP adsorption, and (**i**) Temkin isotherm for P-MP adsorption.

The removal isotherm mechanism was studied to provide information about the attitude of mono-layer adsorption and multi-layer adsorption, in addition to suggesting the nature of the adsorption surface and interactions between the nano-sorbent and the adsorbate^37^ and are shown in Fig. 12. Therefore, the Langmuir (Fig. 12a,d,g), Freundlich (Fig. 12b,e,h), and Temkin isotherm models (Fig. 12c,f,i) were identified by examining the adsorptive capacity of SnO_2_ NPs, and they are expressed by (Eq. 5), (Eq. 6) and (Eq. 7) and are shown in Fig. 12a–i, respectively.

5 $$\frac{1}{{{\text{q}}}_{{\text{e}}}}= \frac{1}{{{\text{q}}}_{\mathrm{ max}}{{\text{K}}}_{{\text{L}}}}\frac{1}{{{\text{C}}}_{{\text{e}}}}+ \frac{1}{{{\text{q}}}_{\mathrm{ max}}}$$

6 $${{\text{logq}}}_{{\text{e}}}=\mathrm{log }{{\text{K}}}_{{\text{F}}}+ \frac{1}{{\text{n}}}\mathrm{ log}{{\text{C}}}_{{\text{e}}}$$

7 $${{\text{q}}}_{{\text{e}}}={{\text{B}}}_{{\text{T}}}{{\text{lnK}}}_{{\text{T}}}+{{\text{B}}}_{{\text{T}}}{{\text{lnC}}}_{{\text{e}}}$$

where the q_max_ represents maximum mono-layer adsorption capacity (mg of phenolic compounds per g of SnO_2_ NPs). R_L_ is _the_ separation factor, and *n* is the surface heterogeneity index, whereas K_L_ K_F_, and B_T_ are the Langmuir constant (L/mg), the Freundlich constant (mg^1/n^ g^−1^ L^−1/n^), and the Temkin constant (J/mol).

As illustrated in Fig. 12, the R^2^ values were lower in the Langmer and Ferndlich models plots than in the Temkin model. Therefore, all PHE, P-NP, and P-MP adsorption processes were fitted well to Temkin isoherm model. The adsorption isotherm results of PHE, P-NP, and P-MP indicate that multi-layer adsorption occurred. Furthermore, with an increase in nano-sorbent surface coverage, the heat of the adsorption system (all molecules) in the layer decreases linearly rather than logarithmically^38^.

According to Temkin isoherm, higher B_T_ values refer to stronger adsorption binding between the adsorbent and adsorbate; thus, the phenolates are more strongly attracted to the SnO_2_ NPs surface. Meanwhile, higher K_T_ values refer to a higher concentration of phenolic molecules on the SnO_2_ NPs surface, confirming a favorability extent of adsorption. Overall, these results indicate that the uniform distribution of phenolate groups on the adsorbate and SnO_2_ NPs surfaces may cause a uniform distribution of binding energies^39,40^.

All isotherm parameters of the Langmuir, Freundlich and Temkin models were provided in Table 2.

**Table 2:** Calculated isotherm parameters for PHE, P-NP, and P-MP adsorption.

Isotherm model	Parameters	Adsorbate		
		PHE	P-NP	P-MP
Langmuir	*q*_*max*_ (mg/g)	0.674	0.891	0.595
	*K*_*L*_ (L/mg)	3.737	1.247	1.820
	*R*_*L*_ (dimensionless)	0.098	0.211	0.224
	*R*^2^	0.479	0.752	0.267
Freundlich	*K*_*F*_ (mg^1–1/n^ g^−1^ L^−1/n^)	7.156 × 10^–10^	1.247 × 10^–4^	1.808 × 10^–3^
	1/*n* (dimensionless)	0.240	0.653	0.173
	*R*^2^	0.685	0.818	0.595
Temkin	BT (J mol^−1^)	57.16	26.894	139.030
	KT (L mg^−1^)	4.425	3.834	4.306
	R^2^	0.884	0.986	0.990

### Adsorption thermodynamics

**Figure 13:**
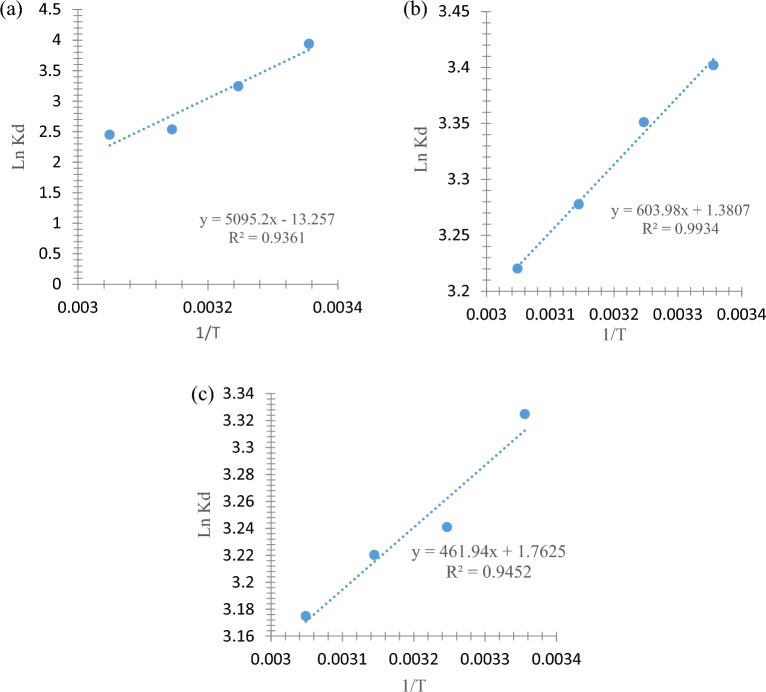
Thermodynamics plots of (**a**) PHE, (**b**) P-NP, and (**c**) P-MP removal.

Thermodynamic adsorption was studied to calculate the amount of energy and heat changing during the PHE, P-NP, and P-MP adsorption processes and to determine these processes' spontaneity as shown in Fig. 13. So, the dissociation coefficient (*K*_d_) and standard Gibbs free energy (ΔG^0^) were calculated at a specific temperature employing Eqs. (8), (9), and plots of ln *K*_d_ vs. 1/T were produced as depicted in Fig. 13. In addition, the standard enthalpy changes (ΔH^0^), and standard entropy changes (ΔS^0^) were calculated from van't Hoff plot and Eq. (10).

8 $${K}_{d}= \frac{{q}_{e}}{{C}_{e}}$$

9 $$\Delta {{\text{G}}}^{0}= \Delta {{\text{H}}}^{0}- \Delta {{\text{S}}}^{0}{\text{T}}$$

10 $${{\text{lnK}}}_{{\text{d}}}= \frac{\Delta {{\text{S}}}^{0}}{{\text{R}}}- \frac{\Delta {{\text{H}}}^{0}}{{\text{RT}}}$$

Where the R is ideal gas constant, and T represents temperature in the Kelvin scale.

Regarding the data in Table 3, the ∆G^o^ negative values suggest the PHE (Fig. 13a), P-NP (Fig. 13b) and P-MP(Fig. 13c) adsorption processes were spontaneous and favorable. This corresponds to the fact that adsorption decreases with high temperatures. A negative ΔH^0^ value indicates an exothermic nature for all PHE, P-NP and P-MP adsorption processes. Moreover, the negative ΔS^0^ (randomness index in adsorption system) value in PHE adsorption indicates that the system is becoming more ordered. In contrast, in P-NP and P-MP adsorption thermodynamics, the positive value of ΔS^0^ indicates the processes favored to be less ordered. This may be justified by the fact that the degree of the freedom of solvent molecules decreases as the number or size of surrounding molecules increases, imposing less ordered adsorption^41^. In general, further understanding of thermodynamic parameters can help to promote the adsorption process for several applications.

**Table 3:** Thermodynamic parameters for the PHE, P-NP, and P-MP adsorption.

Adsorbate	ΔG^0^ (kJ·mol^−1^)				ΔH^0^ (kJ·mol^−1^)	ΔS^0^ (J·mol^−1^·K^−1^)
	T (Kelvin)					
	298	308	318	328		
PHE	− 9.760	− 8.305	− 6.709	− 6.677	− 42.361	− 110.22
P-NP	− 8.429	− 8.581	− 8.666	− 8.782	− 5.021	11.479
P-MP	− 8.237	− 8.299	− 8.514	− 8.658	− 0.437	1.666

#### Comparison with other sorbent to removal of phenolic pollutants

The performance of various adsorbents for phenolic pollutants removal was compared and summarized in Table 4. As can be seen, the removal efficiency of phenolic compounds on SnO_2_ NPs was higher than other adsorbents previously reported in the literature. The SnO_2_ NPs in this study were superior to other adsorbents in terms of large adsorption surface area, adsorption rate, and chemical saving, thereby promoting adsorption capacity in SnO_2_ NPs.

**Table 4:** Comparison of the phenolic compounds removal by SnO_2_ NPs with other sorbents.

Adsorbate	Adsorbent	Removal				References
		Adsorbent dosage (g)	Adsorbate dosage (ppm)	Time (min)	Efficiency (%)	
Phenol	Al_2_O_3_ NPs	0.50	400	120	92	^10^
2-Chlorophenol	Fungus Bio-Sorbent	0.10	100	360	23	^42^
*p*-nitrophenol	C[4]BS	0.10	7	60	58	^34^
Phenol	AC–Fe_2_O_3_ NPs	0.20	2	120	90.5	^32^
Phenol	AC–TiO_2_ NPs	0.20	2	120	89.5	
Phenol	DPDQ	0.10	3	15	76.1	^33^
*p*-nitrophenol P-NP	SnO_2_ NPs	0.10	3	30	85	
Phenol PHE	SnO_2_ NPs	0.20	3	30	95	This work
*p*-Methoxyphenol P-MP	SnO_2_ NPs	0.10	3	30	94	

#### Molecular docking studies

**Figure 14:**
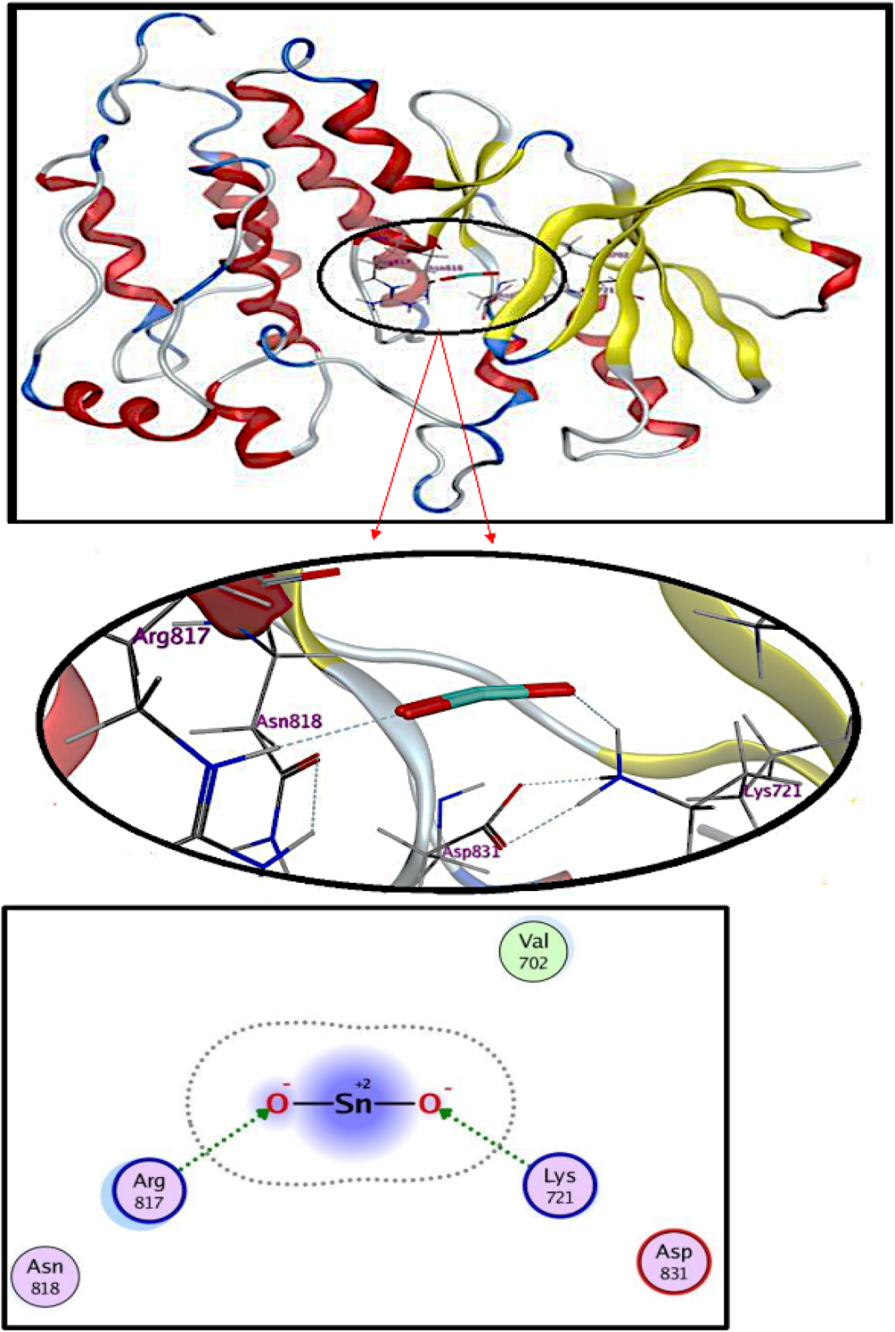
2D and 3D view of the binding interaction of SnO_2_ NPs within active sites of the EGFR receptor (PDB: 4HJO).

Molecular docking studies of synthesized nanomaterials revealed their possible interactions with active site residues of given protein targets^40^. The SnO_2_ NPs demonstrated moderate binding energies against the crystal structure of the inactive EGFR tyrosine kinase domain in complex with erlotinib (PDB: 4HJO) showing their critical interaction with key amino acids. Docked complexes obtained for SnO_2_ showed two conventional hydrogen bonds with ARG817 (2.3 Å) and LYS721(2.0 Å) with a binding score of − 6.619 kcal/mol. The negative and low docking score value indicate that the compounds underwent strong and favorable binding interactions. Our findings are compatible with those found in the literature^43^, and RMSD refine 1.1985 Å is less than 2 Å, i.e., a good result, as depicted in Fig. 14. While in silico investigations have exhibited encouraging outcomes concerning the capability of SnO_2_ NPs to impede the EGFR tyrosine kinase pathway, additional inquiries are imperative to ascertain their therapeutic effectiveness in clinical applications. This research holds the potential to advance the development of efficacious cancer therapies, underscoring the necessity for comprehensive investigations to validate the therapeutic utility of these NPs as anticancer agents targeting the EGFR tyrosine kinase pathway^44^.

## Conclusion

In summary, we report here the green synthesis of the SnO_2_ NPs using *O. basilicum* leaves extracts. Fabricated SnO_2_ NPs showed superior PHE, P-NP, and P-MP removal from the aqueous system. The influence of five key variables on all PHE, P-NP, and P-MP adsorption by fabricated SnO_2_ NPs, including pH, starting PHE, P-NP, and P-MP concentrations, nano-sorbent dose, agitation time, and temperature was also investigated and enhanced. According to the data, the maximum removal effectiveness of PHE, P-NP, and P-MP were 95%, 85%, and 94%, respectively. Based on the Kinetic experiments, the PHE, P-NP, and P-MP adsorption data demonstrated the best fit by the pseudo-2nd order kinetic model, and all mechanisms are controlled by chemisorption. In equilibrium studies, adsorption processes are described by Temkin isotherm model. The Thermodynamic results show that the PHE, P-NP, and P-MP adsorption processes onto fabricated SnO_2_ NPs have a spontaneous and exothermic nature.

Based on the overall analysis, it can be indicated that the eco-friendly approach serves as an invaluable tool for the production of nano-sorbents mediated by plant extracts. Moreover, our nano-sorbent's ultra-effective adsorption of phenolic pollutants may be attributed mainly to the availability of active adsorption sites regardless of their extremely small size. Our molecular docking studies revealed the potential of SnO_2_ NPs to interact with the EGFR tyrosine kinase domain and inhibit its activity, suggesting a novel anticancer mechanism of action. However, we need more research and progress into nanoscale adsorbents and their development for real applications.

## Data Availability

The datasets generated during and/or analysed during the current study are available from the corresponding author on reasonable request.

## References

[1] Mohammed BB, Yamni K, Tijani N, Alrashdi AA, Zouihri H, Dehmani Y, Chung I-M, Kim S-H, Lgaz H (2019). Adsorptive removal of phenol using faujasite-type Y zeolite: Adsorption isotherms, kinetics and grand canonical Monte Carlo simulation studies. J. Mol. Liq..

[2] Nayak PS, Singh BK (2007). Removal of phenol from aqueous solutions by sorption on low cost clay. Desalination.

[3] Abu-Nada A, Abdala A, McKay G (2021). Removal of phenols and dyes from aqueous solutions using graphene and graphene composite adsorption: A review. J. Environ. Chem. Eng..

[4] Almasi A, Dargahi A, Amrane A, Fazlzadeh M, Mahmoudi M, Hashemian A (2014). Effect of the retention time and the phenol concentration on the stabilization pond efficiency in the treatment of oil refinery wastewater. Fresenius Environ. Bull..

[5] Juturu R, Murty VR, Selvaraj R (2024). Efficient adsorption of Cr (VI) onto hematite nanoparticles: ANN, ANFIS modelling, isotherm, kinetic, thermodynamic studies and mechanistic insights. Chemosphere.

[6] Pradeep N, Anupama S, Navya K, Shalini H, Idris M, Hampannavar U (2015). Biological removal of phenol from wastewaters: A mini review. Appl. Water Sci..

[7] Yu P, Huang K, Zhao J, Zhang C, Xie K, Deng F, Liu H (2010). A novel separation technique: Gas-assisted three-liquid-phase extraction for treatment of the phenolic wastewater. Sep. Purif. Technol..

[8] Sultan M, Siddique M, Khan R, Fallatah AM, Fatima N, Shahzadi I, Waheed U, Bilal M, Ali A, Abbasi AM (2022). Ligustrum lucidum leaf extract-assisted green synthesis of silver nanoparticles and nano-adsorbents having potential in ultrasound-assisted adsorptive removal of methylene blue dye from wastewater and antimicrobial activity. Materials.

[9] Al-Tawarh WM, Altarawneh RM, Al-Trawneh SA, Alshahateet SF, Al-Taweel S (2023). An effective calix [4] arene-based adsorbent for tetracycline removal from water systems: Kinetic, isotherm, and thermodynamic studies. J. Chem. Res..

[10] Safwat SM, Mohamed NY, Meshref MN, Elawwad A (2022). Adsorption of phenol onto aluminum oxide nanoparticles: Performance evaluation, mechanism exploration, and principal component analysis (PCA) of thermodynamics. Adsorpt. Sci. Technol..

[11] Dehmani Y, Dridi D, Lamhasni T, Abouarnadasse S, Chtourou R, Lima EC (2022). Review of phenol adsorption on transition metal oxides and other adsorbents. J. Water Process Eng..

[12] Nassar NN, Hassan A, Pereira-Almao P (2011). Metal oxide nanoparticles for asphaltene adsorption and oxidation. Energy Fuels.

[13] Vinayagam R, Nagendran V, Goveas LC, Narasimhan MK, Varadavenkatesan T, Chandrasekar N, Selvaraj R (2024). Structural characterization of marine macroalgae derived silver nanoparticles and their colorimetric sensing of hydrogen peroxide. Mater. Chem. Phys..

[14] Xia L, Ju J-G, Xu W, Ding C-K, Cheng B-W (2016). Preparation and characterization of hollow Fe_2_O_3_ ultra-fine fibers by centrifugal spinning. Mater. Des..

[15] Sridevi H, Bhat R, Selvaraj R (2023). Removal of an agricultural herbicide (2, 4-Dichlorophenoxyacetic acid) using magnetic nanocomposite: A combined experimental and modeling studies. Environ. Res..

[16] Jeevanandam J, Barhoum A, Chan YS, Dufresne A, Danquah MK (2018). Review on nanoparticles and nanostructured materials: History, sources, toxicity and regulations. Beilstein J. Nanotechnol..

[17] Nassr-Allah AA, Aboul-Enein AM, Aboul-Enein KM, Lightfoot DA, Cocchetto A, El-Shemy HA (2009). Anti-cancer and anti-oxidant activity of some Egyptian medicinal plants. J. Med. Plants Res..

[18] Valifard M, Mohsenzadeh S, Kholdebarin B (2017). Salinity effects on phenolic content and antioxidant activity of Salvia macrosiphon. Iran. J. Sci. Technol. Trans. A Sci..

[19] Suresh S, Vennila S, Anita Lett J, Fatimah I, Mohammad F, Al-Lohedan HA, Alshahateet SF, Motalib Hossain M, Rafie Johan M (2022). Star fruit extract-mediated green synthesis of metal oxide nanoparticles. Inorganic Nano-Metal Chem..

[20] Bukhari A, Ijaz I, Gilani E, Nazir A, Zain H, Saeed R, Alarfaji SS, Hussain S, Aftab R, Naseer Y (2021). Green synthesis of metal and metal oxide nanoparticles using different plants’ parts for antimicrobial activity and anticancer activity: A review article. Coatings.

[21] Malik AR, Sharif S, Shaheen F, Khalid M, Iqbal Y, Faisal A, Aziz MH, Atif M, Ahmad S, Fakhar-e-Alam M (2022). Green synthesis of RGO-ZnO mediated Ocimum basilicum leaves extract nanocomposite for antioxidant, antibacterial, antidiabetic and photocatalytic activity. J. Saudi Chem. Soc..

[22] Guo Y, Huang W, Chen B, Zhao Y, Liu D, Sun Y, Gong B (2017). Removal of tetracycline from aqueous solution by MCM-41-zeolite A loaded nano zero valent iron: Synthesis, characteristic, adsorption performance and mechanism. J. Hazard. Mater..

[23] Merzouki M, Challioui A, Bourassi L, Abidi R, Bouammli B, El Farh L (2023). In silico evaluation of antiviral activity of flavone derivatives and commercial drugs against SARS-CoV-2 main protease (3CLpro). Moroc. J. Chem..

[24] Cherriet S, Merzouki M, El-Fechtali M, Loukili E, Challioui A, Soulaymani A, Nanadiyanto A, Ibriz M, Elbekkaye K, Ouasghir A (2023). In silico investigation of aristolochia longa anticancer potential against the epidermal growth factor receptor (EGFR) in the tyrosine kinase domain. Moroc. J. Chem..

[25] Fajriyah N, Mugiyanto E, Rahmasari K, Nur A, Najihah V, Wihadi MN, Merzouki M, Challioui A, Vo T (2023). Indonesia herbal medicine and its active compounds for anti-diabetic treatment: A systematic mini review. Moroc. J. Chem..

[26] Faris A, Edder Y, Louchachha I, Lahcen IA, Azzaoui K, Hammouti B, Merzouki M, Challioui A, Boualy B, Karim A (2023). From himachalenes to trans-himachalol: Unveiling bioactivity through hemisynthesis and molecular docking analysis. Sci. Rep..

[27] Tazikeh S, Akbari A, Talebi A, Talebi E (2014). Synthesis and characterization of tin oxide nanoparticles via the Co-precipitation method. Mater. Sci.-Poland.

[28] Rajakumar G, Rahuman AA, Roopan SM, Khanna VG, Elango G, Kamaraj C, Zahir AA, Velayutham K (2012). Fungus-mediated biosynthesis and characterization of TiO2 nanoparticles and their activity against pathogenic bacteria. Spectrochim. Acta Part A Mol. Biomol. Spectrosc..

[29] Elango G, Kumaran SM, Kumar SS, Muthuraja S, Roopan SM (2015). Green synthesis of SnO2 nanoparticles and its photocatalytic activity of phenolsulfonphthalein dye. Spectrochim. Acta Part A Mol. Biomol. Spectrosc..

[30] Nasiri A, Rajabi S, Amiri A, Fattahizade M, Hasani O, Lalehzari A, Hashemi M (2022). Adsorption of tetracycline using CuCoFe2O4@ Chitosan as a new and green magnetic nanohybrid adsorbent from aqueous solutions: Isotherm, kinetic and thermodynamic study. Arab. J. Chem..

[31] Gong T, Zhou Y, Sun L, Liang W, Yang J, Shuang S, Dong C (2016). Effective adsorption of phenolic pollutants from water using β-cyclodextrin polymer functionalized Fe_3_O_4_ magnetic nanoparticles. RSC Adv..

[32] Abussaud B, Asmaly HA, Saleh TA, Gupta VK, Atieh MA (2016). Sorption of phenol from waters on activated carbon impregnated with iron oxide, aluminum oxide and titanium oxide. J. Mol. Liq..

[33] Al-Trawneh SA, Jiries AG, Alshahateet SF, Sagadevan S (2021). Phenol removal from aqueous solution using synthetic V-shaped organic adsorbent: Kinetics, isotherm, and thermodynamics studies. Chem. Phys. Lett..

[34] Dolaksiz YE, Temel F, Tabakci M (2018). Adsorption of phenolic compounds onto calix [4] arene-bonded silica gels from aqueous solutions. Reactive Funct. Polym..

[35] Horsfall M, Spiff AI (2005). Effect of metal ion concentration on the biosorption of Pb2+ and Cd2+ by Caladium bicolor (wild cocoyam). Afr. J. Biotechnol..

[36] Souilem S, El-Abbassi A, Kiai H, Hafidi A, Sayadi S, Galanakis CM (2017). Olive Oil Production Sector: Environmental Effects and Sustainability Challenges.

[37] Lai B, Zhang Y, Chen Z, Yang P, Zhou Y, Wang J (2014). Removal of p-nitrophenol (PNP) in aqueous solution by the micron-scale iron–copper (Fe/Cu) bimetallic particles. Appl. Catal. B Environ..

[38] Foo KY, Hameed BH (2010). Insights into the modeling of adsorption isotherm systems. Chem. Eng. J..

[39] Zhang A, Li X, Xing J, Xu G (2020). Adsorption of potentially toxic elements in water by modified biochar: A review. J. Environ. Chem. Eng..

[40] Zhang F, Lan J, Yang Y, Wei T, Tan R, Song W (2013). Adsorption behavior and mechanism of methyl blue on zinc oxide nanoparticles. J. Nanopart. Res..

[41] Tabakci M (2008). Immobilization of calix [6] arene bearing carboxylic acid and amide groups on aminopropyl silica gel and its sorption properties for Cr (VI). J. Inclusion Phenomena Macrocyclic Chem..

[42] Majiya H, Clegg F, Sammon C (2023). Bentonite-Chitosan composites or beads for lead (Pb) adsorption: Design, preparation, and characterization. Applied Clay Science.

[43] Riaz S, Ikram M, Naz S, Shahzadi A, Nabgan W, Ul-Hamid A, Haider A, Haider J, Al-Shanini A (2023). Bactericidal action and industrial dye degradation of graphene oxide and polyacrylic acid-doped SnO_2_ quantum dots: In silico molecular docking study. ACS Omega.

[44] Boumezzourh A, Ouknin M, Merzouki M, Dabbous-Wach A, Hammouti B, Umoren P, Costa J, Challioui A, Umoren SA, Majidi L (2023). Acetylcholinesterase, tyrosinase, α-Glucosidase inhibition by *Ammodaucus leucotrichus* Coss. & Dur. fruits essential oil and ethanolic extract and molecular docking analysis. Moroc. J. Chem..

