# Author Correction: Catalytic green synthesis of Tin(IV) oxide nanoparticles for phenolic compounds removal and molecular docking with EGFR tyrosine kinase

**DOI:** 10.1038/s41598-024-58478-w

**Published:** 2024-04-04

**Authors:** S. F. Alshahateet, R. M. Altarawneh, W. M. Al-Tawarh, S. A. Al-Trawneh, S. Al-Taweel, K. Azzaoui, M. Merzouki, R. Sabbahi, B. Hammouti, G. Hanbali, S. Jodeh

**Affiliations:** 1 Department of Chemistry, Faculty of Science, Mutah University, Al-Karak, Jordan; 2 Engineering Laboratory of Organometallic, Molecular Materials and Environment, Faculty of Sciences, Sidi Mohamed Ben Abdellah University, 30000 Fez, Morocco; 3 Euro-Mediterranean University of Fes, BP 15, 30070 Fez, Morocco; 4 Morocco Laboratory of Applied Chemistry and Environment (LCAE) Team (ECOMP), Mohamed 1er University, Oujda, Morocco; 5 Higher School of Technology, Ibn Zohr University, P.O. Box 3007, Laayoune, Morocco; 6 Department of Chemistry, An-Najah National University, Nablus, Palestine

Correction to: *Scientific Reports* 10.1038/s41598-024-55460-4, published online 19 March 2024

The original version of this Article contained an error in the Acknowledgements section. The Decision Number was incorrect.

“The authors gratefully acknowledge the deanship of scientific research—Mutah University, Jordan, for supporting this work. This study was funded by a research grant from Mutah University (Decision No. 525/2022) to buy the chemicals and run analysis. The funders had no role in study design, data collection and analysis, decision to publish or preparation of the manuscript.”

now reads:

“The authors gratefully acknowledge the deanship of scientific research—Mutah University, Jordan, for supporting this work. This study was funded by a research grant from Mutah University (Decision No. 2022/580) to buy the chemicals and run analysis. The funders had no role in study design, data collection and analysis, decision to publish or preparation of the manuscript.”

The original Article has been corrected.

